# Personal

**Published:** 1890-02

**Authors:** 


### Personal.

DR. DANIEL LEWIS, President of the Medical Society of the State of New York, Instructor in Surgery in the New York Post-Graduate School of Medicine, etc., has recently, upon invitation of the Faculty, delivered a course of lectures in the Medical Department of the University of Buffalo, upon "The Nature and Treatment of Cancer." These lectures were illustrated, and were a valuable contribution to the knowledge of this interesting subject.

---

DR. G. W. MCPHERSON, of Lancaster, N. Y., was elected President of the Medical Society of Erie County, at the annual meeting held in this city January 14, 1890. This is a deserved recognition of the professional attainments of one of the most capable members of the Society.

---

DR. L. HOWE, of Buffalo, is mentioned as having an eye upon the presidency of the Medical Society of the State of New York; at all events the doctor appears to be manifesting a special interest in the business affairs of the Society this year. We wish him success.

Dr. W. D. Greene was chosen City Health Physician by the Buffalo Board of Health, at its recent annual meeting. If a change was to be made, no more suitable officer could have been selected.

